# Surgical Strategy for the Repair of Acute Type A Aortic Dissection: A Multicenter Study

**DOI:** 10.3390/jcdd10060253

**Published:** 2023-06-09

**Authors:** Francesco Nappi, Sanjeet Singh Avtaar Singh, Ivancarmine Gambardella, Almothana Alzamil, Antonio Salsano, Francesco Santini, Fausto Biancari, Thibaut Schoell, Nicolas Bonnet, Thierry Folliguet, Antonio Fiore

**Affiliations:** 1 Department of Cardiac Surgery, Centre Cardiologique du Nord, 93200 Saint-Denis, France; almothana.md@gmail.com (A.A.); tiboschoell@hotmail.com (T.S.); drnicolasbonnet@free.fr (N.B.); 2 Department of Cardiothoracic Surgery, Royal Infirmary of Edinburgh, Edinburgh EH16 4SA, UK; sanjeetsinghtoor@gmail.com; 3 Department of Cardiothoracic Surgery, Weill Cornell Medicine—New York, Presbyterian Medical Center, New York, NY 10065, USA; icg9002@med.cornell.edu; 4 Division of Cardiac Surgery, Ospedale Policlinico San Martino, DISC Department, University of Genoa, 16126 Genoa, Italy; ant.salsano@gmail.com (A.S.); francesco.santini@unige.it (F.S.); 5 Heart and Lung Center, Helsinki University Hospital, University of Helsinki, 00231 Helsinki, Finland; faustobiancari@yahoo.it; 6 Department of Cardiac Surgery, Hôpitaux Universitaires Henri Mondor, Assistance Publique-Hôpitaux de Paris, 94000 Creteil, France; thierry.folliguet@aphp.fr (T.F.); fioreant7@yahoo.com (A.F.)

**Keywords:** type A acute aortic dissection, ascending aorta replacement, aortic arch repair, total arch replacement procedure, cerebrovascular perfusion

## Abstract

Type A acute aortic dissection is associated with significant morbidity and mortality, with prompt referral imaging and management to tertiary referral centers needed urgently. Surgery is usually needed emergently, but the choice of surgery often varies depending on the patient and the presentation. Staff and center expertise also play a major role in determining the surgical strategy employed. The aim of this study was to compare the early- and medium-term outcomes of patients undergoing a conservative approach extended only to the ascending aorta and the hemiarch to those of patients subjected to extensive surgery (total arch reconstruction and root replacement) across three European referral centers. A retrospective study was conducted across three sites between January 2008 and December 2021. In total, 601 patients were included within the study, of which 30% were female, and the median age was 64.4 years. The most common operation was ascending aorta replacement (*n* = 246, 40.9%). The aortic repair was extended proximally (i.e., root *n* = 105; 17.5%) and distally (i.e., arch *n* = 250; 41.6%). A more extensive approach, extending from the root to the arch, was employed in 24 patients (4.0%). Operative mortality occurred in 146 patients (24.3%), and the most common morbidity was stroke (75, 12.6%). An increased length of ICU admission was noted in the extensive surgery group, which comprised younger and more frequently male patients. No significant differences were noted in surgical mortality between patients managed with extensive surgery and those managed conservatively. However, age, arterial lactate levels, “intubated/sedated” status on arrival, and “emergency or salvage” status at presentation were independent predictors of mortality both within the index hospitalization and during the follow-up. The overall survival was similar between the groups.

## 1. Introduction

Despite the progress achieved by cardiac surgery in the current era, type A acute aortic dissection (TAAAD) persists as a clinicopathological entity burdened by a high lethality. Awareness of the life-threatening nature of the disease alongside the need for obtaining accurate images hae led to faster diagnoses and better perioperative care. However, the operative mortality associated with TAAAD repair is still discouraging and persistently high. Centers with a high volume of patients treated for acute type A aortic dissection and large databases such as the European registries and the International Acute Aortic Dissection Registry report an OM ranging from 18 to 20%. Complications such as renal failure, major neurological lesions, and postoperative bleeding must be added to this result, which substantially reduce the success offered by the surgical repair and undermine the recovery of patients who survive the operation [1,2,3,4,5]. With regard to operative mortality, in patients requiring complex repair for TAAAD, evidence reported from high-volume aortic centers of excellence revealed better outcomes than that reported in the Nordic Consortium for Acute Type A Aortic dissection (NORCAD), the German Registry for Acute Aortic Dissection (GEERADA), and the International Acute Aortic Dissection Registry (IRAD) [3,4,5,6,7,8]. This discrepancy highlights the greater experience developed in the field of aortic surgery by highly specialized working groups in order to guarantee the best postoperative results through the implementation of the best treatment options in specific patient populations [9,10,11].

In this study, we report the multicenter experience of a large number of patients who required proximal and/or distal aortic repair, considering five different grades of clinical status at the time of hospitalization. We sought to investigate which type of TAAAD repair could improve the patients’ early outcomes and whether using a more conservative approach extended only to the hemiarch while preserving the aortic root could reduce the postoperative mortality.

## 2. Methods

This study is part of a research project approved by the Health Research Authority discussed at the Montpellier University Hospital. Patient consent was obtained after the assigned IRB approval number (IRB-MTP_2022_07_202201173) in accordance with the research guidance. This study complies with the Declaration of Helsinki.

### 2.1. Data Extraction

Comprehensive data extraction retrospectively collected from 3 cardiac surgery centers (Centre Cardiologique du Nord, Saint-Denis, France, University Hospital Henri Mondor, Creteil, France, and DISC Department, University of Genoa) were included in a central cardiac database which was analyzed retrospectively by a statistician at Weill Cornell Medicine, New York, Presbyterian Medical Center. The registry retrospectively collated demographic information, as well as pre-and postoperative clinical information, including mortality, for cardiac surgery procedures in patients who developed TAAAD from 2005 to 2021. In summary, the data entered locally by the surgeons were validated in each single center by the database managers before being loaded into a single database. At this stage, further validation was performed, and missing data reports were generated for the primary variables (e.g., EuroSCORE risk factors, patient identifiers, and outcome data). The data were then double-checked and forwarded to the statistician for cleaning and analysis. Duplicate records were removed, transcriptional discrepancies recoded, and clinical and temporal conflicts and missing data resolved through specific queries to local database managers. Since the enrolled patients were operated on at different times, the lengths of the follow-up might differ. However, the great majority of the patients reached 5 years of follow-up.

### 2.2. Patients and Endpoints

The database included a total of 601 patients with their baseline, demographic, and follow-up data that were examined. The inclusion criteria for this study comprised patients with TAAAD or intramural hematoma involving the ascending aorta. Patients were >18 years of age, with the onset of the symptoms within 7 days of surgery and indication for primary surgical repair of acute type A aortic dissection and any other major cardiac surgical procedure concurrent with surgery for TAAAD and retrograde TAAAD, with the lesion primarily located in the descending aorta. Exclusion criteria consisted of previous procedure for TAAAD, concomitant endocarditis, and TAAAD following blunt or penetrating chest trauma.

The primary endpoint of the study consisted in operative mortality defined as 30-day and in-hospital mortality. Secondary endpoints were stroke or cerebrovascular accident resulting in permanent neurologic deficit, spinal cord injury (SCI; paraplegia, paraparesis), respiratory failure requiring tracheostomy, need for new dialysis, intensive care unit (ICU) stay, a composite of major adverse events (MAE) including operative mortality, cerebrovascular accidents, need for dialysis, or need for tracheostomy and late survival.

The primary and secondary endpoints were assessed by means of the segment of the aorta replaced, conservative versus extensive surgery, and the impairment of the patient’s clinical condition at hospital admission requiring surgery. The urgency of the procedure was classified into five categories established on the increasing severity of hemodynamic instability and the need and timing of cardiopulmonary resuscitation. So, we considered the following status: urgent, emergency grade 1, emergency grade 2, salvage grade 1, and salvage grade 2 [12].

### 2.3. Surgical Procedure

Median sternotomy was performed in all cases for access. The surgical procedure regarding the preferred site for cannulation was as per the surgeon’s preference, and in the majority of the patients, was performed in the innominate artery, right femoral artery, axillary artery, or central aortic lumen. The degree of systemic cooling once the patients were positioned for surgery and cardiopulmonary bypass management was also surgeon specific. Antegrade cardioplegia was used primarily, although retrograde cardioplegia was used in patients with concomitant aortic insufficiency or if extensive aortic arch repair was planned. The ascending aorta excision was extended up to the sinotubular junction. The false lumen of the aortic root was cleared of thrombus to permit the visualization of the defect. The anatomy of the root was inspected alongside the aortic valve leaflets. The intima was re-approximated to the adventitia. The commissures were routinely resuspended using 4-0 or 5-0 sutures reinforced with a Teflon pledget over each commissure. The biologic-glue neo-media technique was routinely performed during the reconstruction, while the use of felt was dictated by the individual surgeons’ habits. The proximal anastomosis was performed using a 4-0 or 5-0 polypropylene suture which concomitantly secured the intima to the adventitia. To achieve an uninterrupted external ring of felt reinforcement, felt neo-media or an overlay of horizontal felt-mattress sutures was positioned circumferentially and dictated by the surgeon. The replacement of the aortic root using a composite valve graft or valve-sparing root reimplantation was recommended in patients with sinus of Valsalva exceeding 4.5 cm in diameter on computed tomography imaging, in the presence of connective tissue disorders, or when the intimal tear involved the sinus. Patients with normal-sized aortic roots but poor-quality valve leaflets underwent concomitant aortic valve replacement, with the use of conventional xenograft or mechanical prosthesis favored.

Total arch replacement procedures (TARP) were performed using deep hypothermic circulatory arrest and with either antegrade or retrograde cerebral perfusion and systemic cooling (19 °C to 25 °C, as per surgeon). Symmetric brain cooling and warming were monitored through the use of near-infrared spectroscopy. The techniques of cerebral protection, type of cannulation, and perfusion were at the discretion of the surgeon. In the majority of the patients, antegrade cerebral protection was delivered using the endoluminal technique or the direct cannulation of the right axillary artery, innominate trunk, or common carotid. The flow rate of the injection was 800–1000 mL/min at 28 °C or 36 °C, while maintaining the systemic pressure between 40 and 60 mmHg. In the remaining 19.5% of the cases undergoing arch repair, the procedure was performed using retrograde cerebral perfusion during deep hypothermic circulatory arrest. Cerebroplegia was administered by a cannula inserted into the superior vena cava and delivered at 200–350 mL/min at 18 °C with the central venous pressure maintained between 25 and 35 mmHg.

One- and four-branch prostheses were preferred in patients undergoing TARP. This comprised the resection of all the aortic tissue up to the left common carotid artery (total hemiarch) or the reimplantation of the innominate trunk only (partial hemi-arch). TARP that required large vessel reimplantation was instead performed in patients with a large arch aneurysm or an extensive intimal lesion within the arch. The surgical option to perform arch debranching and selective vessel implantation was preferred in patients with connective tissue disorders or significant dislocation of the great vessels. Patients who needed a frozen elephant trunk (FET) procedure underwent either insular replantation or selective debranching/implantation of vessels. An antegrade cardiopulmonary bypass was reinstituted using a reperfusion branch of the graft. Systemic warming was performed while preserving a temperature gradient of 10 °C between the blood and the core temperature during hemostasis. The remaining anastomoses were completed and reinforced according to the previously described technique, and the cardiopulmonary bypass was stopped when the core body temperature reached 36 °C.

### 2.4. Statistical Analysis

#### Descriptive Statistics—Continuous Variables

The categorical variables are reported as numbers and percentages and were compared with the Pearson’s χ^2^ test or the Fisher’s exact test, as appropriate. The continuous variables are presented as median and interquartile range and were compared with the *t*-test or the Mann–Whitney U test after checking for normal distribution with the Shapiro–Wilk test.

The Kruskal–Wallis H test was applied to determine if there were statistically significant differences between two or more groups for an independent variable or a continuous or ordinal dependent variable.

The pre/intra/post-operative variables were compared across subgroups of our population sample to eminently discern their association with the extent of aortic replacement (i.e., “ascending only”, “+root”, “+arch”, “+arch & root”) and with the urgency status at presentation (i.e., “urgent”, “emergency 1”, “emergency 2”, “salvage 1”, “salvage 2”)^11^. Survival was assessed with Kaplan–Meier curves log-rank tests to compare the groups. For all statistical analyses, a *p*-value < 0.05 was accepted as significant without adjustment for multiple testing.

Risk Adjustment—Risk factors for mortality, both during index hospitalization and during follow-up, according to published evidence and clinical experience, were evaluated first with univariate regression. Predictors presenting an association, with *p* < 0.2, with in-hospital or follow-up mortality were retained, respectively, for the multivariable logistic and the Cox regression models. Multi-collinearity among independent variables was assessed with variance inflation factor analysis and excluded by a value ≤3.

Statistical software—RSudio version 4.0.3, with the packages “dplyr, ggfortify, ggplot2, ranger, survival, survminer” was utilized for the statistical elaboration.

## 3. Results

**Figure 1:**
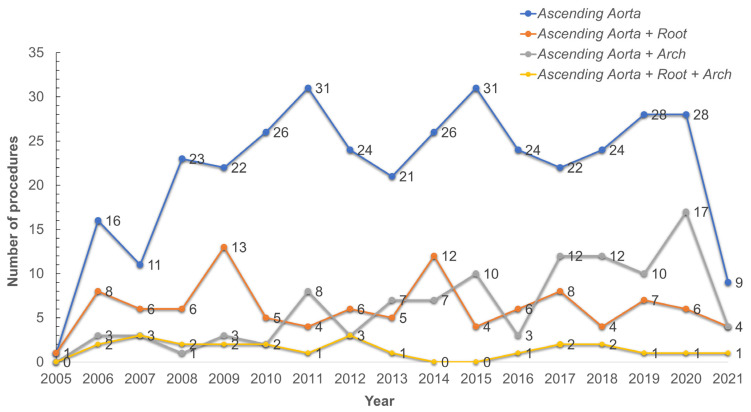
Title—Yearly volume of type A aortic dissection repairs. Yearly volume of type A aortic dissection repairs. The curves are color-coded according to the aortic segment replaced.

A total of 601 patients who underwent surgical repair for TAAAD in three hospitals from 2008 to 2021 were identified (median age 64.4 [median interquartile (IQR) 20.1], 30% female). All patients had at least one follow-up visit, and the median follow-up time was 29 months (IQR; 79 months). We observed an increase in the number of cases performed annually over the years until 2019, as shown in Figure 1. However, a reduction in the procedure was recorded in the period 2019–2021 corresponding to the pandemic crisis due to COVID-19.

### 3.1. Overall Sample

The pre/intra/post-operative variables of the overall sample are presented in Table 1 The subjects who underwent the greatest number of TAAAD repair procedures were males in the sixth decade of life. Approximately half of the patients received surgery for TAAAD in an urgent setting, while the remaining half consisted of patients who underwent an emergency or salvage procedure. The aortic valve was affected by moderate to severe regurgitation in more than a third of the cases. Malperfusion was observed in one-quarter of the patients, with the brain being the most affected site. The most common operation was ascending aorta replacement (*n* = 246, 40.9%). The aortic repair was extended proximally (i.e., root *n* = 105; 17.5%) and distally (i.e., arch *n* = 250; 41.6%). A more extensive approach, extending from the root to the arch, was employed in 24 patients (4.0%). Operative mortality occurred in 146 patients (24.3%), and the most common morbidity was stroke (75, 12.6%, Table 1).

### 3.2. Subgroup Analysis I: Aortic Segments

The patients who underwent the more conservative approach that included only ascending aortic replacement were, on average, 8 years older than patients who received the most extensive repair that included root and/or arch extension. We observed that preoperative clinical and biochemical risk factors were similar between subgroups. Presumably, bicuspid aortic valves and the occurrence of aortic regurgitation were more common in patients undergoing root replacement/repair. The extent of repair did not influence operative mortality and major adverse events, except for the stroke rate, which was the lowest in the “+root” group (7.6%) compared to the “+arch” group (21.9%) (*p* = 0.01). The incidences of stroke, cord injury, tracheostomy, and renal failure requiring dialysis were not statistically different nor was the occurrence of major adverse events (Table 2).

### 3.3. Subgroup Analysis II: Conservative vs. Extensive Surgery

Most patients received a conservative surgery (*n* = 393, 65.4%) with hemiarch surgery (*n* = 147, 24.4%), followed by an interposition graft (*n* = 246, 40.9%). Extensive surgery (*n* = 208, 34.6%) included root replacement (*n* = 105, 17.5%) and total arch repair (*n* = 103, 17.1%), and this trend was constant over time. Patient characteristics, operative data, and outcomes in the overall sample and according to the extent of surgical repair and urgent status are presented in Table 3.

The conservative group was more likely to be older (66.9 [ median IQR, 20.4] vs. 61.1 [median IQR, 17.4] years; *p* < 0.01) with more comorbidities, such as pulmonary disease (6.6% vs. 3.4%; *p* = 0.14), extracardiac arteriopathy (4.6% vs. 1.4%; *p* = 0.08), poor mobility (9.9% vs. 4.8%; *p* = 0.04), and worse hemodynamic conditions (51.7% vs. 45.3%; *p* = 0.10). The extensive group was more likely to be male, with recent myocardial infarction and bicuspid aortic valve disease. The incidence of aortic regurgitation (AR) was higher in the conservative group that was more likely to reveal mild and moderate AR (39.0% vs. 15.4% and 17.6% vs. 12.5%; *p* < 0.01), while the extensive group recorded more severe AR (6.9% vs. 43.3%; *p* < 0.001). The rates of malperfusion and rupture as emerged in salvage 2 status were similar (Table 3)

### 3.4. Subgroup Analysis II: Urgency Status

While the demographics did not vary across the cohorts, the biochemical markers were significantly impacted by the urgency status. For instance, the creatinine levels and hemoglobin levels mirrored the progression in the status at presentation, from “urgent” (Cr. 82 mg/dL, Hb 116 g/dL) to “salvage 2” (Cr 145 mg/dL, Hb 99 g/dL) (*p* < 0.01). The clinical preoperative risk factors and the aortic valve status were similar across the groups. Malperfusion was directly associated with the level of urgency: while it was absent in the “urgent” and “emergency 1” groups (0.0%), it was present at the highest level in the “emergency 2” group (79.5%), (*p* < 0.01). The incidences of stroke, chordal injury, and tracheostomy were not statistically significant across the cohorts, whereas renal failure requiring dialysis was significantly associated with “emergency 2” (18.6%) and “salvage 1” (12.5%), (*p* < 0.01). Operative mortality was significantly associated with the urgency status: “urgent”, 15.8%, “emergency 1”, 25.2%, “emergency 2”, 34.8%, “salvage 1”, 50.0%, and “salvage 2”, 60.0% (*p* < 0.01). The composites of major adverse events were present at a higher level in “emergency 2” (50.9%), “salvage 1” (66.7%), and “salvage 2” (60.0%), (*p* < 0.01, Table 4).

### 3.5. Survival

**Figure 2:**
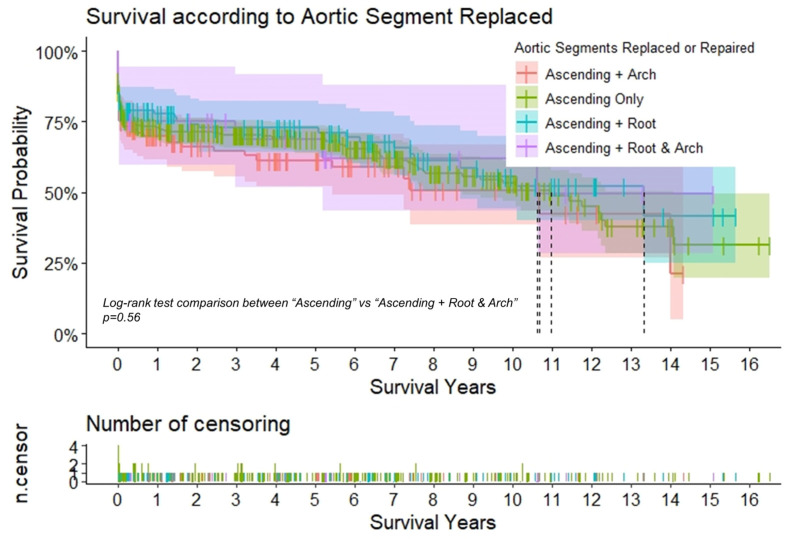
Survival according to the aortic segments replaced. Kaplan–Meier curves to assess survival after type A aortic dissection repair. The curves are color-coded according to the aortic segment replaced, and the relative shaded areas represent the 95% confidence interval. The censored patients are represented by the short vertical lines along the survival curves. The dotted black lines represent the estimated median survival, which could only be calculated if the survival had dropped by <50% for the relative subgroup at the end of the study period.

**Figure 3:**
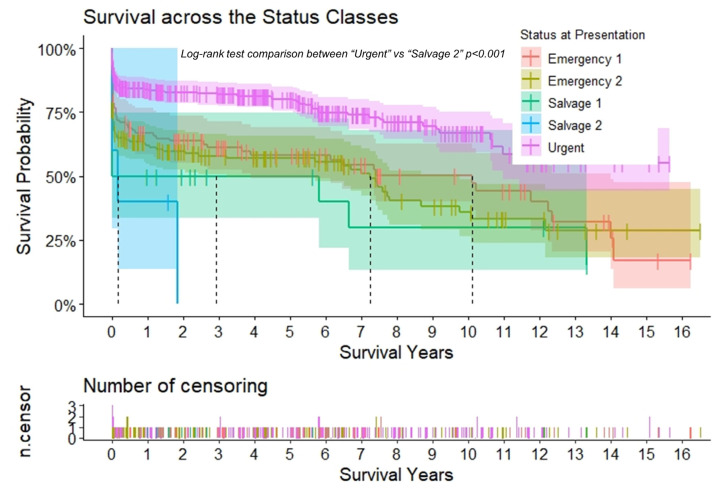
Survival according to the urgency status. Kaplan–Meier curves to assess survival after type A aortic dissection repair. The curves are color-coded according to the urgency status at presentation, and the relative shaded areas represent the 95% confidence interval. The censored patients are represented by the short vertical lines along the survival curves. The dotted black lines represent the estimated median survival, which could only be calculated if the survival had dropped <50% for the relative subgroup at the end.

In the overall sample, survival was 73.3% ± 1.8 at 1 year and 68.2% ± 2.0 at 5 years (Table 5). The extent of aortic repair did not affect the survival (Table 5, Figure 2). Survival was significantly altered by the urgency status at presentation. At the 1-year mark, the survival of the “urgent” group was more than double the survival of the “salvage 2” group (84.0% ± 2.1 vs. 40.0% ± 21.9) (*p* < 0.01). At the 5-year mark, the survival was 80.2% ± 2.4 and 50% ± 10.2, respectively, for the “urgent” and “salvage 1” groups (Table 5, Figure 3). In Table 6, adverse events by center are reported.

### 3.6. Predictors of In-Hospital and Follow-Up Mortality

Age, arterial lactate levels, “intubated/sedated” status on arrival, and “emergency or salvage” status at presentation were independent predictors of mortality both within the index hospitalization and during the follow-up. In addition, while in-hospital mortality was predicted by a “poor mobility” status, mortality during follow-up was higher in patients with congenital bicuspid aortic valves. Both in-hospital and follow-up mortality in univariate logistic regression analyses for cerebral perfusion had odds ratios of 0.37 and 0.07, while root or arch replacements conferred hazard ratios of 0.98 and 0.99, respectively. The predictors that presented an association characterized by *p* < 0.2 were entered into the multivariable models (Table 7).

## 4. Discussion

Although the ideal repair strategy for TAAAD should aim to an extensive treatment with minimal risk of late reoperation, surgical-related mortality in combination with the severity of the patient’s clinical condition may guide the surgeon towards a different surgical strategy. The achievement of the goal of reduced operative mortality in patients presenting with an additional surgical complexity can be hindered by an increase in operating times and by the secondary risk of prolonged periods of organ and cardiac ischemia. These conditions may greatly affect the acceptable operational mortality in those patients whose hospitalization occurs in salvage 1 and 2 clinical status.

Given the evidence gathered in multicenter studies, this concept seems to guide the choice of the surgical option of many surgeons. The report of the Society of Thoracic Surgeons from 2014 to 2016 indicates the operative mortality of extended TAR procedures was 26.9% compared to 16.3% for hemiarch repair [12,13]. Likewise, complete resection of the intimal tear and prosthetic replacement of the ascending aorta with or without hemiarch were still the most commonly performed procedures for type A aortic dissections, as reported in large series of patients [14,15,16,17]. There are no unanimous results from the literature. Proponents of the conservative repair argue that the limited involvement of arch repair was associated with lower operative mortality, thereby restoring the functional integrity of the aorta [18,19]. Advocates of extensive surgery in addition to ascending aortic replacement cite the adverse consequences of the persistent false recirculating lumen and argue that in patients with impaired optimal organ perfusion, more extensive repair may prevent a progressive state of adverse multiorgan ischemia and reduce the risk of reoperation [20,21,22,23].

A look into the literature reveals conflicting data. A meta-analysis of 2221 patients by Poon et al. demonstrated no difference in mortality or long-term reintervention/reoperation rates for patients who underwent hemiarch and total arch repair, with the mortality rate ranging from 3.60 to 24.1% for hemiarch replacement patients and from 3.85 to 28.57% for total arch replacement patients [24]. These data are consistent with the results of the present study. Another meta-analysis compared proximal repair to extended arch repair, reporting that the former involved a reduced risk of early mortality (relative risk, 0.69) albeit showing a higher risk of distal reoperations (relative risk, 3.14) [25]. The conflicting results were even more evident in single-center studies. Rylski and colleagues [19] observed an increase in operative mortality with the progressive extension of surgical repair involving all or part of the aortic arch (ascending only, 9.8%; hemiarch, 21.6%; TAR, 28.6%). Kim and colleagues, instead, reported results that did not match previous results when comparing patients undergoing TAR to those who received hemiarch repair (13.4% vs. 9.7%). However, they observed a significantly higher incidence of permanent neurologic deficit in the TAR group than in the group where the procedure was limited to the ascending aorta alone with extension to the hemiarch (22.7% vs. 6.3%) [26].

A large body of available literature reports disparities in the rates observed in several studies, with neutral findings for TAAAD repair. Di Eusanio and colleagues [27] compared patients receiving a conservative repair with those undergoing total arch replacement procedures and observed that both the incidence of operative mortality (24.1% vs. 22.6%) and that of cerebro-vascular accident with permanent neurological deficit (9.1% vs. 7.5%) were quite similar. Likewise, Zhang and colleagues [28] reported similar results comparing conservative hemiarch repair with frozen elephant trunk, and the difference in mortality ranged from 5.4% to 5.7%, while that in permanent neurologic deficit ranged from 1.4% to 2.3%. A reduced operative mortality consistently below 10% stands out among advocates when comparing a more conservative surgical approach to aggressive techniques such as TARP, antegrade stent grafting, or FET. In fact, this low mortality seems to be the prerogative of a few centers.

In this analysis, it was observed that to limit the operative mortality without incurring the risk of reoperation and compromising the late survival, a conservative approach was used for TAAAD repair. We reported a lower number of operations with a more extensive approach involving the aortic root and aortic arch in low-risk patients; however, the limited extent of the operation did not expose them to a great risk of future reoperations. The cohort of patients who received a conservative procedure consisted of older subjects with multiple comorbidities, such as pulmonary disease, extracardiac arteriopathy, poor mobility, and emergency status. This group received either ascending aortic replacement associated with root sparing and aortic valve resuspension (40.9%) or hemiarch replacement (24.4%). The patients for whom a more extensive approach was used were younger, with a greater risk for subsequent reoperations, and revealed a higher incidence of bicuspid aortic valve, a slightly higher incidence of recent myocardial infarction (*p* = 0.34), and more severe aortic regurgitation (*p* < 0.01). The choice to minimalize the procedure of aortic repair was dictated by their stable clinical condition at hospitalization for TAAAD, associated with the manifestation of more limited comorbidities. This cohort of patients received an urgent procedure and underwent surgery on a working day following hospitalization or later during the index hospitalization [12]. In these paucisymptomatic subjects, hemodynamic parameters were steady, with no evidence of clinical signs of malperfusion and/or aortic rupture. So, this status may have allowed them to better tolerate the longer cross-clamp and bypass times required to complete larger repairs. The total arch replacement was performed in patients with a tear localized to the aortic arch, in those who suffered a rupture in the presence of a large arch aneurysm, or in subjects who experienced bicuspid aortic valve disease. We reported only 17.5% of patients who received aortic root replacement. The choice to perform this procedure was dictated by the extension of the tear in the Valsalva sinus, the severe dilatation of the aortic root, or the presence of a bicuspid aortic valve. A considerable proportion of these patients had severe aortic regurgitation and were referred to the surgeon for emergency procedure 2 surgery, which was performed immediately after hospital admission or in consideration of the worsening of the patient’s clinical condition. These patients revealed hemodynamic instability despite the use of inotropes and/or malperfusion; however, they did not require cardiopulmonary resuscitation. However, caution was dictated for patients disclosing root or arch aneurysms of more moderate size, and the choice of a conservative approach was preferred especially for those in advanced age or with extensive comorbidities or hemodynamic instability.

**Figure 4:**
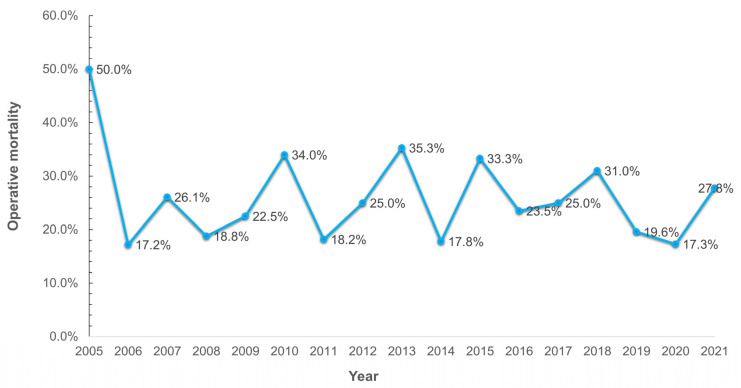
Operative mortality during the study period. The line graph shows the operative mortality resulting from the sum of the percentages of cases over the years (2005–2021).

We noted that the strategy used to manage aortic repair in TAAAD did not produce differences in operative mortality between the groups (*p* = 0.84). The raw operative mortality rate was 24.3%, with a decrease over time from 34.3% in 2010 to 27.8% in 2021 (Figure 4), albeit a substantial correlation with the severity of the clinical conditions at hospital admission was noted. We observed a significant difference in operative mortality with respect to the urgency of the required procedure based on the patient’s clinical condition (from 15.8% in the urgent group to 60% in the salvage 2 group). However, the operative mortality was comparable to those reported in other national and international registries. The crude mortality rate reported in the UK nationwide dataset for type A acute aortic dissection (UK National Adult Cardiac Surgical Audit) was 17.8%, decreasing over time from 22% in 2009 to 15% in 2018 [2]. Previous reports from IRAD revealed a decrease in surgical mortality from 25% to 18%. This reduction means that, over time, the in-hospital mortality rate of TAAAD patients dropped significantly from 31% to 22% [15]. In the last report from IRAD that assembled information from 2952 subjects with type A aortic dissection enrolled from 28 referral centers in North America, Europe, Asia, and Australia, the overall mortality rate for TAAAD in hospital admission was 5.8% at 48 h. A non-surgery cohort TAAAD revealed a mortality rate of 0.5% per hour (23.7% at 48 h), which decreased the mortality rate to 4.4% in the surgery cohort at 48 h [5]. The results from the NORCAAD, a collaborative mortality registry of eight academic cardiothoracic centers in Denmark, Finland, Iceland, and Sweden, recorded an in-hospital mortality rate of 16% [3]. Likewise, the GERAADA collected data on 2137 recipients of a surgical procedure for TAAAD enrolled in 50 centers in Germany, Austria, Switzerland, and Luxembourg from July 2006 to June 2017, scoring an overall 30-day mortality of 16.9% [3,18]. However, conflicting results in mean operative mortality have been reported in single-center studies that pointed out consistent inequalities of numerical percentages [9,10,11] with respect to registry studies [1,2,3,4,5]. For example, recently, Lau and colleagues [20] observed a mean operative mortality of 5.6%, with no substantial difference between patients who received a conservative repair involving root-sparing or hemiarch surgery and those undergoing a more aggressive surgery with an extended repair involving root replacement and/or a full aortic arch.

In the present analysis, similar results were also reported with respect to individual complications. The incidence of permanent and temporary neurological damage was equally distributed in both groups, supporting our preferred cerebral protection strategy adopting normothermic cerebral perfusion and ensuring a very brief deep hypothermic circulatory arrest in the low body. Although there was a relatively high incidence of preoperative malperfusion in both groups (24.9% vs. 23.6%; *p* = 0.78), we observed that permanent sequelae were rare and were in part due to our favored primary aortic repair approach supported by the rapid restoration of true-lumen blood flow, preferentially through cannulation of the innominate artery into the central true lumen. The composite MAE outcomes (37.7% vs. 44.2%; *p* = 0.14) were higher in the “extensive” group and were probably dictated by the reflex effect more dependent on the preoperative comorbidity than by the surgical procedure. In univariable analyses, extensive surgery including root or arch replacement, cerebral perfusion, and recent myocardial infarction was not a predictor of operative mortality, while malperfusion, creatine levels, and increase of cardiac biomarkers reached statistical significance. In contrast, for both groups, multivariate logistic regression showed that age, arterial lactate level, intubated/sedated status at hospitalization, as well as the degree of severity determined by the emergency or salvage status were predictive of a higher operative mortality. Although patients receiving the extensive surgical approach incurred the expected longer operative times, a careful preoperative selection allowed them to contain this additional risk and led to a gratifying early and late survival rate. The survival rates at 1 year (72.8% vs. 70.0%), 5 years (68.6% vs. 61.3%), and 10 years (53.4% vs. AR < 10) were similar in both groups (*p* = 0.56).

There is a wide variety of procedures to manage aortic arch repair in the context of TAAAD, including ascending aortic replacement extended to hemiarch replacement, antegrade stent grafting, and more complex surgical solutions such as TARP or TARP with FET. In our analysis, we found that hemiarch replacement is a durable technique with a low risk of reoperation if it is addressed to selected patients who do not present with arch aneurysmal disease or a bicuspid aortic valve. In the present study, the results reported after arch repair suggest that in patients with BAV or arch aneurysm disease, TARP can be performed safely, with no difference in operative mortality compared to other procedures, especially in younger patients with few comorbidities (*p* = 0.37). Although both large databases [13] and single-center results have shown an increase in mortality [19] and permanent neurological deficit [26] in patients undergoing extensive arch repair, we observed that adequate patient selection patient, cerebral protection, and meticulous attention to surgical hemostasis have a favorable impact on patients’ recovery by reducing major morbidity and mortality to levels comparable with those associated with less complex repairs.

Total arch replacement associated with the frozen elephant trunk procedure is another arch repair strategy that deserves reflection. Centers of excellence, strong proponents of this remediation strategy, have reported impressive results. Sun and colleagues performed this procedure in 148 patients with TAAAD associated with arch injury, arch aneurysm, or Marfan syndrome, reporting exceptional results. They observed no differences in in-hospital mortality (4.7% vs. 6.1%; *p* = 0.74), SCI (1.4% vs. 0; *p* = 0.1), or stroke (2.7% vs. 1.5%; *p* = 0.1) with respect to patients who underwent the replacement of the hemiarch. Furthermore, the risk of false lumen thrombosis was considerably reduced, and fewer reoperations were reported in the FET group (1 vs. 4 patients; *p* = 0.03). However, despite improved aortic remodeling, there was no difference in late mortality [8]. Shrestha and colleagues have routinely used the frozen elephant trunk procedure in patients with TAAAD as well. Operative mortality was 13%, similar to the operative mortality reported with many other procedures. However, in this series, a stroke rate of 15% and an incidence of spinal cord injury of 5% emerged, which are worrying, particularly if 12% of the patients discharged from the hospital after surgery still needed aortic reoperations afterwards [29]. Deserving attention is spinal cord injury, which has been highlighted as a particularly devastating complication associated with the FET procedure. A large meta-analysis based on a high number of TAAAD patients undergoing FET revealed an aggregated SCI rate of 4.7%, which increased significantly when a stent longer than 15 cm was implanted or when coverage of the aorta was extended to the T8 or lower segments [23]. Poon and colleagues evaluated the results of a database consisting of 507 patients receiving arch repair, reporting that FET was associated with a higher risk of spinal cord injury compared to the conventional TARP conventional procedure (1.5% vs. 3.9%; *p* = 0.03) [30].

In this series, we reported similar rates of SCI in recipients of a conservative and an extended approach (3.3% vs. 5.8%; *p* = 0.22) and in patients hospitalized with more or less severe clinical conditions (2.6% urgency vs. 5.6% emergency 1 and emergency 2; *p* = 0.35). Furthermore, for TARP associated with FET, we described a procedure characterized by a very short time of circulation arrest in the lower part of the body, which was kept at temperatures that never dropped below 28 °C. The true lumen was rapidly perfused after making the distal anastomosis, followed by the execution of the proximal aortic and cerebral arterial trunk anastomoses [31]. Gambardella and colleagues observed that spinal cord injury after elective and open thoracoabdominal aorta aneurysm repair for chronic type A dissection is a rare complication [32]. The indication for a frozen elephant trunk procedure is questionable considering the limited percentage of patients requiring a late distal reoperation [8,19,20], and therefore caution is warranted in recommending the routine use of frozen elephant trunk. Nevertheless, it would be advantageous to evaluate a select group of patients receiving the FET procedure, whom we expect to be at high risk for distal reoperation.

Patients with TAAAD referred for root replacement undergo a more complex operation, demanding notably longer bypass and cross-clamp times than those receiving root replacement. The fragility characteristic of the dissected aortic tissue could constitute an additional risk factor for the reimplantation of the coronary button, and its manipulation could be dangerous. Furthermore, these patients are at a higher risk of both postoperative bleeding and ischemia, which can be life-threatening. However, in centers of high experience and volume of cases treated, it has been observed that a selection of patients can mitigate the greater risks dictated by the surgical strategy in the cohort of TAAAD patients requiring elective aortic root surgery [33]. In this series, 21.5% of the patients who tended to have more severe BAV and aortic regurgitation underwent root replacement via a biological or mechanical composite graft or valve-sparing aortic replacement. We did not observe an increase in operative mortality compared with patients receiving a root-sparing procedure (19% versus 24.3%; *p* = 0.37). It is important to observe, however, that root-sparing surgery is performed in most patients with TAAAD even when moderate aortic regurgitation is present. In our series, 56.6% of the patients in the root preservation group had mild or moderate AR, while severe AR (44.3%) was treated in 90 of 129 patients who underwent root replacement. Importantly, in most of the patients, the valvular dysfunction was primarily due to the collapse of one or more commissures of the aortic valve rather than related to a true native valvular disease. This group of patients presenting with severe AR also had aortic root dilatation. We believe that the scrupulous resuspension of the commissures plays a key role in restoring the correct geometry of the aortic root, thus allowing to re-establish the diameter of the sinotubular junction and guaranteeing the resolution of severe aortic regurgitation with the continence of the aortic valve. We performed an aortic suture corresponding exactly to the upper limit of the commissures, recording a complete resolution of the AR in the group of patients receiving conservative surgery, and we observed that this technique is safe and durable. A word of caution is directed to choosing composite conduits toward the valve-sparing root replacement procedure as the best definitive treatment option for root pathology in the context of TAAAD. We performed the valve-sparing root replacement procedure in selected cases (1.3%) that included young subjects with intact aortic flap tissue. Sievers and colleagues reported higher rates of patients undergoing valve-sparing root replacement with excellent short- and medium-term outcomes [10].

## 5. Limitations

A limitation of this study is that it excluded patients who were managed conservatively or who did not survive admission to a tertiary referral center. This remains an issue with most retrospective studies in aortic dissection, which prevents the comparison of the characteristics of these patients to those of patients who were operated. Furthermore, regional variations in practice such as intensive care management, postoperative management, transfusion thresholds, and medications used were not accounted for. The retrospective nature of this study also resulted in the variable distribution of the surgical strategies (arch repair vs. no-arch repair). Because of its multicenter nature, this study was also biased by surgeon-specific and center-specific preferences including cannulation and neuroprotective strategies. Other variables not included comprise the socioeconomic demographics of the patients which were variable between the centers. The patients presented different lengths of follow-up, implying that few reached 10 years of follow-up.

## 6. Conclusions

TAAADs present with a variety of manifestations; however, there were no significant differences in surgical mortality between patients managed with extensive surgery and those managed conservatively. An increased length of ICU admission was noted in the extensive surgery group who included younger and more frequently male patients. The overall survival was similar between patients undergoing conservative and extensive surgical procedures. Further studies inclusive of patients who were managed conservatively should be conducted to permit the comparison of risk factors and adverse outcomes. Ongoing follow-up is needed to identify if the long-term outcomes of extensive surgery are superior to those of conservative surgery.

## Figures and Tables

**Table 1:** Title—Pre/intra/post-operative variables in the overall sample.

Pre/Intra/Post-Operative Variables in the Overall Sample			
Demographics		Malperfusion—*n* (%)	147 (24.5)
❖ Age (years)—median (IQR)	64.4 (20.1)	❖ Cerebral—*n* (%)	80 (13.3)
❖ BMI (kg/m^2^)—median (IQR)	25.8 (5.2)	❖ Spinal—*n* (%)	12 (2.0)
❖ Female—*n* (%)	180 (30.0)	❖ Renal—*n* (%)	61 (10.1)
Biochemistry		❖ Mesenteric—*n* (%)	33 (5.5)
❖ Creatinine (mg/dL)—median (IQR)	88.4 (29.1)	❖ Peripheral—*n* (%)	32 (5.3)
❖ Hemoglobin (g/dL)—median (IQR)	121.0 (28.5)		
❖ Platelet Count (×109 L)—median (IQR)	220.0 (194.5)	Aortic Segments Replaced	
❖ Arterial Lactate (mmol/L)—median (IQR)	2.2 (2.3)	❖ Ascending only—n (%)	246 (62.6)
		❖ Hemi-Arch—n (%)	147 (37.4)
❖ Cardiac Biomarkers Increase—*n* (%)	150 (25.0)	❖ Ascending + Root—*n* (%)	105 (17.5)
Comorbidities and Presentation		❖ Ascending + Arch—*n* (%)	103 (17.5)
❖ Diabetes—*n* (%)	36 (6.0)		
❖ Cerebro-vascular accident—*n* (%)	32 (5.3)		
❖ Pulmonary disease—*n* (%)	33 (5.5)	Type of Root Procedure	
❖ Extracardiac arteriopathy—*n* (%)	21 (3.5)	❖ Modified Bentall—*n* (%)	121 (20.1)
❖ Poor mobility—*n* (%)	49 (8.2)	❖ David Procedure—*n* (%)	5 (0.8)
❖ Moderate-to-severe frailty—*n* (%)	3 (0.5)	❖ Yacoub Procedure—*n* (%)	3 (0.5)
❖ Recent myocardial infarction—*n* (%)	19 (3.2)	❖ Type of Arch Procedure	
❖ Preoperative cardiac massage—*n* (%)	26 (4.3)		
Status		❖ Total Arch—*n* (%)	52 (8.7)
❖ Urgent—*n* (%)	304 (50.6)	❖ Total Arch + FET—*n* (%)	51 (8.5)
❖ Emergency 1—*n* (%)	107 (17.8)	❖ Type of Cerebroplegia	
❖ Emergency 2—*n* (%)	161 (26.8)	❖ Antegrade—*n* (%)	248 (41.3)
❖ Salvage 1—*n* (%)	24 (4.0)	❖ Retrograde *n* (%)	117 (19.5)
❖ Salvage 2—*n* (%)	5 (0.8)	❖ Adverse Events	
Aortic Valve		❖ Stroke—*n* (%)	76 (12.6)
❖ Bicuspid—*n* (%)	12 (2.0)	❖ Spinal cord injury—*n* (%)	25 (4.2)
❖ Regurgitant		❖ Tracheostomy—*n* (%)	27 (4.5)
❖ No trace—*n* (%)	203 (33.8)	❖ Hemodialysis—*n* (%)	63 (10.5)
❖ Mild—*n* (%)	185 (30.8)	❖ Operative mortality—*n* (%)	146 (24.3)
❖ Moderate—*n* (%)	95 (15.8)	❖ Major Adverse Events—*n* (%)	240 (39.9)
❖ Severe—*n* (%)	117 (19.5)	❖ ICU Stay (days)—median (IQR)	9.0 (17.0)

Abbreviations and acronyms: BMI = body mass index; ICU = intensive care unit; IQR = interquartile range; major adverse events = composite of in-hospital mortality and stroke, spinal cord injury, tracheostomy, and hemodialysis.

**Table 2:** Pre/intra/post-operative variables after type A aortic dissection repair, according to the aortic segment replaced.

Pre/Intra/Post-Operative Variables according to Aortic Segment Replaced.					
No. of Patients	Ascending Only N^o^ 246	+Root N^o^ 105	+Arch N^o^ 250	+Root & Arch N^o^ 24	*p* Value
Demographic Characteristics					
❖ Age (years)—median (IQR)	67.4 (20.6)	61.7 (19.8)	61.1 (16.5)	59.7 (11.4)	<0.01
❖ BMI (kg/m^2^)—median (IQR)	25.9 (5.4)	25.6 (4.3)	25.6 (4.5)	25.4 (4.0)	0.64
❖ Female—*n* (%)	128 (34.9)	23 (21.9)	25 (23.8)	4 (16.7)	<0.01
Biochemistry					
❖ Cr (mg/dL)—median (IQR)	88.5 (32.4)	88.0 (19.9)	88.4 (33.0)	81.0 (7.0)	0.50
❖ Hb (g/dL)—median (IQR)	122.0 (28.5)	122.0 (31.0)	120.0 (28.0)	112.5 (29.5)	0.16
❖ PLT (×10^9^ L)—median (IQR)	211.0 (166.0)	250.0 (182.5)	199.0 (224.0)	281.0 (186.2)	0.05
❖ Lactate (mmol/L)—median (IQR)	2.2 (2.4)	2.1 (2.0)	2.0 (2.4)	2.5 (1.9)	0.80
❖ Enzyme Increment—*n* (%)	90 (24.5)	81 (77.1)	32 (30.5)	20 (83.3)	0.41
Comorbidities and Presentation					
❖ Diabetes—*n* (%)	21 (5.7)	6 (5.7)	9 (8.6)	0 (0.0)	0.42
❖ Cerebro-vascular accident—*n* (%)	19 (5.2)	7 (6.7)	5 (4.8)	1 (4.2)	0.91
❖ Pulmonary disease—*n* (%)	25 (6.8)	5 (4.8)	1 (1.0)	2 (8.3)	0.12
❖ Extracardiac arteriopathy—*n* (%)	18 (4.9)	1 (1.0)	2 (1.9)	0 (0.0)	0.12
❖ Poor mobility—*n* (%)	37 (10.1)	7 (6.7)	5 (4.8)	0 (0.0)	0.12
❖ Moderate-to-severe frailty—*n* (%)	3 (0.8)	0 (0.0)	0 (0.0)	0 (0.)	0.59
❖ Recent myocardial infarction—*n* (%)	9 (2.5)	6 (5.7)	3 (2.9)	1 (4.2)	0.40
❖ Cardiac massage—*n* (%)	14 (3.8)	6 5.7)	6 (5.7)	0 (0.0)	0.51
❖ Intubated/sedated—*n* (%)	100 (27.2)	32 (30.5)	37 (35.2)	10 (41.7)	0.23
Status					0.06
❖ Emergency 1—*n* (%)	77 (21.0)	18 (17.1)	12 (11.4)	0 (0.0)	
❖ Emergency 2—*n* (%)	98 (26.7)	29 (27.6)	30 (28.6)	4 (16.7)	
❖ Salvage 1—*n* (%)	12 (3.3)	6 (5.7)	6 (5.7)	0 (0.0)	
❖ Salvage 2—*n* (%)	4 (1.1)	1 (1.0)	0 (0.0)	0 (0.0)	
❖ Urgent—*n* (%)	176 (48.0)	51 (48.6)	57 (54.3)	20 (83.3)	
Aortic Valve					
❖ Bicuspid—*n* (%)	4 (1.1)	6 (5.7)	1 (1.0)	1 (4.2)	0.02
❖ Regurgitant—*n* (%)					<0.01
○ No trace—*n* (%)	135 (36.9)	10 (9.5)	57 (54.3)	1 (4.2)	
○ Mild—*n* (%)	147 (40.2)	8 (7.6)	29 (27.6)	1 (4.2)	
○ Moderate—*n* (%)	59 (16.1)	17 (16.2)	12 (11.4)	7 (29.2)	
○ Severe—*n* (%)	25 (6.8)	70 (66.7)	7 (6.7)	15 (62.5)	
Malperfusion	89 (24.3)	27 (25.7)	27 (25.7)	4 (16.7)	0.81
❖ Cerebral—*n* (%)	47 (12.8)	15 (14.3)	16 (15.2)	2 (8.3)	
❖ Spinal—*n* (%)	7 (1.9)	3 (2.9)	2 (1.9)	0 (0.0)	
❖ Renal—*n* (%)	37 (10.1)	11 (10.5)	12 (11.4)	1 (4.2)	
❖ Mesenteric—*n* (%)	25 (6.8)	4 (3.8)	3 (2.9)	1 (4.2)	
❖ Peripheral—*n* (%)	18 (4.9)	5 (4.8)	7 (6.7)	2 (8.3)	
Outcomes					
❖ Stroke—*n* (%)	43 (11.7)	8 (7.6)	23 (21.9)	2 (8.3)	0.01
❖ Spinal cord injury—*n* (%)	12 (3.3)	6 (5.7)	6 (5.7)	1 (4.2)	0.57
❖ Tracheostomy—*n* (%)	16 (4.4)	6 (5.7)	5 (4.8)	0 (0.0)	0.67
❖ Dialysis—*n* (%)	33 (9.0)	11 (10.5)	15 (14.3)	4 (16.7)	0.35
❖ Operative mortality—*n* (%)	89 (24.3)	20 (19.0)	31 (29.5)	6 (25.0)	0.37
❖ Major Adverse Events—*n* (%)	137 (37.3)	40 (38.1)	53 (50.5)	10 (41.7)	0.11
❖ ICU Stay (days)—median (IQR)	7.0 (15.0)	10.0 (22.0)	11.0 (17.0)	15.5 (13.2)	<0.01

Abbreviations and acronyms: BMI = body mass index; Cr = creatinine; Hb = hemoglobin; ICU = intensive care unit; IQR = interquartile range; lactate = arterial lactate; major adverse events = composite of in-hospital mortality and stroke, spinal cord injury, tracheostomy, and hemodialysis; PLT = platelets count; enzymes = cardiac enzymes.

**Table 3:** Pre/intra/post-operative variables after type A aortic dissection repair, comparing a conservative (i.e., ascending ± hemiarch replacement) to an extensive (i.e., +aortic root or total aortic arch replacement) surgical approach.

Pre/Intra/Post-Operative Variables			
No. of Patients	Conservative N^o^ 393	Extensive N^o^ 208	*p* Value
Demographic Characteristics			
❖ Age (years)—median (IQR)	66.9 (20.4)	61.1 (17.4)	<0.01
❖ BMI (kg/m^2^)—median (IQR)	25.9 (5.6)	25.7 (4.3)	0.53
❖ Female—*n* (%)	137 (34.9)	43 (20.7)	<0.01
Biochemistry			
❖ Cr (mg/dL)—median (IQR)	88.4 (31.6)	88.0 (25.7)	0.86
❖ Hb (g/dL)—median (IQR)	122.0 (29.0)	119.0 (27.0)	0.22
❖ PLT (×10^9^ L)—median (IQR)	212.5 (168.0)	230.0 (204.7)	0.12
❖ Lactate (mmol/L)—median (IQR)	2.2 (2.3)	2.3 (2.2)	0.85
❖ Enzymes Increase—*n* (%)	95 (24.2)	55 (26.4)	0.61
Comorbidities and Presentation			
❖ Diabetes—*n* (%)	22 (5.6)	14 (6.7)	0.71
❖ Stroke—*n* (%)	7 (1.8)	7 (3.4)	0.35
❖ Pulmonary disease—*n* (%)	26 (6.6)	7 (3.4)	0.14
❖ Extracardiac arteriopathy—*n* (%)	18 (4.6)	3 (1.4)	0.08
❖ Poor mobility—*n* (%)	39 (9.9)	10 (4.8)	0.04
❖ Moderate-to-severe frailty—*n* (%)	3 (0.8)	0 (0.0)	0.59
❖ Recent myocardial infarction—*n* (%)	10 (2.5)	9 (4.3)	0.34
❖ Cardiac massage—*n* (%)	14 (3.6)	12 (5.8)	0.29
❖ Intubated/sedated—*n* (%)	107 (27.2)	72 (34.6)	0.07
Status			0.10
❖ Emergency 1—*n* (%)	79 (20.1)	28 (13.5)	
❖ Emergency 2—*n* (%)	108 (27.5)	53 (25.5)	
❖ Salvage 1—*n* (%)	12 (3.1)	12 (5.8)	
❖ Salvage 2—*n* (%)	4 (1.0)	1 (0.5)	
❖ Urgent—*n* (%)	190 (48.3)	114 (54.8)	
Aortic Valve			
❖ Bicuspid—*n* (%)	4 (1.0)	8 (3.8)	0.04
❖ Regurgitant—*n* (%)			<0.01
○ No/trace—*n* (%)	143 (36.5)	60 (28.8)	
○ Mild—*n* (%)	153 (39.0)	32 (15.4)	
○ Moderate—*n* (%)	69 (17.6)	26 (12.5)	
○ Severe—*n* (%)	27 (6.9)	90 (43.3)	
Malperfusion	98 (24.9)	49 (23.6)	0.78
❖ Cerebral—*n* (%)	52 (13.2)	28 (13.5)	
❖ Spinal—*n* (%)	8 (2.0)	4 (1.9)	
❖ Renal—*n* (%)	41 (10.4)	20 (9.6)	
❖ Mesenteric—*n* (%)	26 (6.6)	7 (3.4)	
❖ Peripheral—*n* (%)	22 (5.6)	10 (4.8)	
Outcomes			
❖ Stroke—*n* (%)	48 (24.9)	28 (23.6)	0.75
❖ Spinal cord injury—*n* (%)	13 (3.3)	12 (5.8)	0.22
❖ Tracheostomy—*n* (%)	18 (4.6)	9 (4.3)	1
❖ Dialysis—*n* (%)	37 (9.4)	26 (12.5)	0.30
❖ Operative mortality—*n* (%)	97 (24.7)	49 (23.6)	0.84
❖ Major Adverse Events—*n* (%)	148 (37.7)	92 (44.2)	0.14
❖ ICU Stay (days)—median (IQR)	8.0 (15.0)	11.0 (22.0)	0.03

Abbreviations and acronyms: BMI = body mass index; Cr = creatinine; Hb = hemoglobin; ICU = intensive care unit; IQR = interquartile range; lactate = arterial lactate; major adverse events = composite of in-hospital mortality and stroke, spinal cord injury, tracheostomy, and hemodialysis; PLT = platelets count; enzymes = cardiac enzymes.

**Table 4:** Pre/intra/post-operative variables after type A aortic dissection repair, according to the urgency status at presentation.

Pre/Intra/Post-Operative Variables according to Urgency Status at Presentation						
Variables	Urgent N^o^ 304	Emergency 1 N^o^ 107	Emergency 2 N^o^ 161	Salvage 1 N^o^ 24	Salvage 2 N^o^ 5	*p* Value
Demographic Characteristics						
❖ Age (years)—median (IQR)	64.2 (18.2)	64.0 (21.2)	63.4 (21.2)	67.1 (13.1)	73.2 (13.2)	0.41
❖ BMI (kg/m^2^)—median (IQR)	25.8 (5.4)	26.8 (5.0)	25.7 (4.7)	25.3 (3.2)	24.2 (2.1)	0.41
❖ Female—*n* (%)	104 (34.2)	26 (24.3)	43 (26.7)	6 (25.0)	1 (20.0)	0.23
Biochemical Analysis						
❖ Cr (mg/dL)—median (IQR)	82.0 (23.0)	94.1 (40.2)	96.0 (40.5)	88.0 (27.0)	145.0 (34.5)	<0.01
❖ Hb (g/dL)—median (IQR)	116.0 (25.0)	130.0 (26.0)	125.5 (28.0)	114.0 (18.0)	99.0 (28.0)	<0.01
❖ PLT (×10^9^ L)—median (IQR)	254.0 (187.0)	198.0 (67.5)	207.5 (182.2)	166.0 (208.0)	128.0 (23.0)	<0.01
❖ Lactate (mmol/L)—median (IQR)	2.5 (2.5)	1.2 (1.3)	2.3 (2.0)	2.3 (4.5)	3.3 (6.0)	<0.01
❖ Enzymes Increase—*n* (%)	82 (27.0)	14 (13.1)	41 (25.5)	12 (50.0)	1 (20.0)	<0.01
Patients Status Before Surgery						
❖ Diabetes—*n* (%)	23 (7.6)	4 (3.7)	7 (4.3)	2 (8.3)	0 (0.0)	0.46
❖ Cerebro-vascular accident—*n* (%)	13 (4.3)	6 (5.6)	11 (6.8)	1 (4.2)	1 (20.0)	0.46
❖ Pulmonary disease—*n* (%)	17 (5.6)	8 (7.5)	6 (3.7)	1 (4.2)	1 (20.)	0.42
❖ Extracardiac arteriopathy—*n* (%)	6 (2.0)	8 (7.5)	6 (3.7)	1 (4.2)	0 (0.0)	0.12
❖ Poor mobility—*n* (%)	33 (10.9)	5 (4.7)	9 (5.6)	2 (8.3)	0 (0.0)	0.16
❖ Moderate-to-severe frailty—*n* (%)	2 (0.7)	0 (0.0)	1 (0.6)	0 (0.0)	0 (0.0)	0.93
❖ Recent myocardial infarction—*n* (%)	7 (2.3)	3 (2.8)	7 (4.3)	2 (8.3)	0 (0.0)	0.44
❖ Cardiac massage—*n* (%)	0 (0.0)	0 (0.0)	0 (0.0)	21 (87.5)	5 (100)	<0.01
❖ Intubated/sedated—*n* (%)	95 (31.2)	15 (14.0)	59 (36.6)	9 (37.5)	1 (20.0)	<0.01
Aortic Valve						
❖ Bicuspid—*n* (%)	3 (1.0)	4 (3.7)	5 (3.1)	0 (0.0)	0 (0.0)	0.30
❖ Regurgitant						0.14
○ No trace—*n* (%)	110 (36.2)	27 (25.2)	55 (34.4)	10 (41.7)	1 (20.0)	
○ Mild—*n* (%)	96 (31.6)	36 (33.6)	47 (29.4)	5 (20.8)	1 (20.0)	
○ Moderate—*n* (%)	36 (11.8)	27 (25.2)	27 16.9)	3(12.5)	2 (40.)	
○ Severe—*n* (%)	62 (20.4)	17 (15.9)	31 (19.4)	6 (25.0)	1 (20.0)	
Malperfusion	0 (0.0)	0 (0.0)	128 (79.5)	16 (66.7)	3 (60.0)	<0.01
❖ Cerebral—*n* (%)	0 (0.0)	0 (0.0)	66 (41.0)	12 (50.0)	2 (40.0)	
❖ Spinal—*n* (%)	0 (0.0)	0 (0.0)	8 (5.0)	4 (16.7)	0 (0.0)	
❖ Renal—*n* (%)	0 (0.0)	0 (0.0)	55 (34.2)	4 (16.7)	2 (40.0)	
❖ Mesenteric—*n* (%)	0 (0.0)	0 (0.0)	25 (15.5)	5 (20.8)	3 (60.0)	
❖ Peripheral—*n* (%)	0 (0.0)	0 (0.0)	30 (18.6)	2 (8.3)	0 (0.0)	
Aortic Segments Replaced						0.06
❖ Ascending Only—*n* (%)	176 (57.9)	77 (72.0)	98 (60.9)	12 (50.0)	4 (40.0)	
❖ +Root—*n* (%)	51 (16.8)	18 (16.8)	29 (18.0)	6 (25.0)	1 (20.0)	
❖ +Arch—*n* (%)	57 (18.8)	12 (11.2)	30 (18.6)	6 (25.0)	0 (0.0)	
❖ +Root & Arch—*n* (%)	20 (6.6)	0 (0.0)	4 (2.5)	0 (0.0)	0 (0.0)	
❖ Adverse Events						
❖ Stroke—*n* (%)	41 (13.5)	11 (10.3)	18 (11.2)	6 (25.0)	0 (0.0)	0.28
❖ Spinal Cord Injury—*n* (%)	8 (2.6)	6 (5.6)	9 (5.6)	2 (8.3)	0 (0.0)	0.35
❖ Tracheostomy—*n* (%)	16 (5.3)	3 (2.8)	8 (5.0)	0 (0.0)	0 (0.0)	0.63
❖ Dialysis—*n* (%)	24 (7.9)	6 (5.6)	30 (18.6)	3 (12.5)	0 (0.0)	<0.01
❖ Operative mortality—*n* (%)	48 (15.8)	27 (25.2)	56 (34.8)	12 (50.0)	3 (60.0)	<0.01
❖ Major Adverse Events—*n* (%)	100 (32.9)	39 (36.4)	82 (50.9)	16 (66.7)	3 (60.0)	<0.01
❖ ICU Stay (days)—median (IQR)	11.0 (20.0)	5.0 (10.0)	8.0 (16.0)	3 (13.5)	1.0 (14.0)	<0.01

Abbreviations and acronyms: BMI = body mass index; Cr = creatinine; Hb = hemoglobin; ICU = intensive care unit; IQR = interquartile range; lactate = arterial lactate; major adverse events = composite of in-hospital mortality and stroke, spinal cord injury, tracheostomy, and hemodialysis; PLT = platelets count; enzymes = cardiac enzymes.

**Table 5:** Survival after type A aortic dissection. Survival after type A aortic dissection repair in the overall sample and across subgroups according to the aortic segment replaced and according to the urgency status at presentation.

Survival—Overall Sample						
Elapsed Time	At Risk—N^o^	Events—N^o^	Survival—%	SE—%		
1-Year	370	157	73.3	1.8		
5-Year	225	22	68.2	2		
10-Year	69	33	53.5	2.8		
Survival According to Aortic Segments Repaired						
Elapsed Time	Ascending	+Root	+Arch	+Root & Arch	*p* value *	
1-Year—% ± SE	72.8 ± 2.4	80.0 ± 4.0	70.0 ± 4.5	75.0 ± 8.8		
5-Year—± SE	68.6 ± 2.5	72.7 ± 4.5	61.3 ± 5.2	68.7 ± 10.1	NS	
<0.01						
Elapsed Time	Urgent	Emergency 1	Emergency 2	Salvage 1	Salvage 2	*p* value ^$^
1-Year—% ± SE	84.0 ± 2.1	66.9 ± 4.6	62.1 ± 3.8	50 ± 10.2	40.0 ± 21.9	
5-Year—% ± SE	80.2 ± 2.4	58.3 ± 5.0	56.9 ± 4.0	50 ± 10.2	At Risk < 10	<0.01

Abbreviations and acronyms: NS = not significant; SE = standard error. * Log-rank test comparison between “Ascending” vs. “+ Root & Arch”. ^$^ Log-rank test comparison between “Urgent” vs. “Salvage 2”.

**Table 6:** Summary of events. Summary of mortality, morbidity, major and minor adverse events by center are reported.

Adverse Events for Center					
	Total	Center CN	Center MR	Center GA	*p* Value
❖ In-Hospital Mortality	146 (24.3)	42 (25.1)	79 (27.0)	25 (17.7)	0.10
❖ Stroke	76 (12.6)	37 (22.2)	33 (11.3)	6 (4.3)	<0.01
❖ Need of Tracheostomy	27 (4.5)	4 (2.4)	17 (5.8)	6 (4.3)	0.23
❖ Need of Dialysis	63 (10.5)	4 (2.4)	49 (16.7)	10 (7.1)	<0.01
❖ Spinal Cord Injury	25 (4.2)	10 (6.0)	13 (4.4)	2 (1.4)	0.13
❖ Major Adverse Events	240 (39.9)	66 (39.5)	134 (45.7)	40 (28.4)	<0.01

**Table 7:** Variables associated with hospital or follow-up mortality were inserted into the multivariable logistic regression and Cox regression models, respectively. The estimates express odds ratios for in-hospital mortality and hazard ratios for follow-up mortality.

(A) Univariable predictors of operative mortality and follow-up mortality in patients who underwent repair for acute type A aortic dissection				
Univariate Logistic Regression				
Predictors	Mortality			
	Operative		Follow-Up	
Age (years)	<0.01		<0.01	
Body Mass Index (Kg/m^2^)	0.43		0.84	
Female	0.37		0.24	
Creatinine (g/dL)	<0.01		<0.01	
Hemoglobin (mg/dL)	0.07		0.15	
Platelet Count (×109 L)	0.13		0.46	
Arterial Lactate (mmol/L)	<0.01		<0.01	
Cardiac Biomarkers Increase	<0.01		0.48	
Diabetes	0.61		0.6	
Prior CVA	0.35		0.14	
Pulmonary disease	0.01		<0.01	
Extracardiac arteriopathy	0.64		0.16	
Poor mobility	0.15		0.27	
Moderate-to-severe frailty	0.13		0.32	
Recent myocardial infarction	0.02		0.02	
Preoperative cardiac massage	<0.01		<0.01	
Intubated/sedated at arrival	<0.01		0.11	
Status: Emergency or Salvage	<0.01		<0.01	
Bicuspid Aortic Valve	0.46		0.14	
Aortic Regurgitation	0.65		0.53	
Malperfusion	<0.01		<0.01	
Cerebral Perfusion	0.37		0.07	
Root or Arch replaced	0.98		0.99	
(B) Multivariable predictors of operative mortality and follow-up mortality in patients who underwent repair for acute type A aortic dissection				
Predictor	Estimate	95% Confidence Interval		*p* Value
		Lower Limit	Upper Limit	
(A) Operative Mortality				
Age (years)	1.04	1.02	1.06	<0.01
Creatinine (g/dL)	1	0.99	1.01	0.17
Hemoglobin (mg/dL)	0.99	0.98	1.01	0.51
Platelet Count (×109 L)	1	0	1	0.57
Arterial Lactate (mmol/L)	1.37	1.2	1.58	<0.01
Cardiac Biomarkers Increment	1.14	0.64	1.98	0.65
Pulmonary disease	2.06	0.74	5.52	0.15
Poor mobility	3.03	1.2	7.52	0.02
Moderate-to-severe frailty	1.58	0.11	39.77	0.73
Recent myocardial infarction	1.81	0.54	5.67	0.31
Preoperative cardiac massage	1.3	0.45	3.69	0.62
Intubated/sedated at arrival	2.74	1.65	4.59	<0.01
Emergency or Salvage	2.6	1.32	5.14	<0.01
Malperfusion	1.02	0.53	2	0.93
(A) Follow-Up Mortality				
Age (years)	1.03	1.01	1.04	<0.01
Creatinine (g/dL)	1	0.99	1	0.07
Hemoglobin (mg/dL)	0.99	0.99	1	0.53
Arterial Lactate (mmol/L)	1.21	1.14	1.29	<0.01
Cerebro-vascular accident	1.47	0.8	2.7	0.21
Pulmonary disease	1.7	0.94	3.05	0.07
Extra-cardiac arteriopathy	0.92	0.45	1.9	0.83
Recent myocardial infarction	1.05	0.53	2.09	0.88
Preoperative cardiac massage	1.29	0.68	2.44	0.43
Intubated/sedated at arrival	1.68	1.21	2.32	<0.01
Emergency or Salvage	2.04	1.37	3.04	<0.01
Bicuspid Aortic Valve	2.7	1.23	5.92	0.01
Malperfusion	0.82	0.54	1.25	0.37

## Data Availability

Nappi, Gambardella, Salsano, and Fiore had full access to all of the data in the study and take responsibility for the integrity of the data and the accuracy of the data analysis. The data underlying this article will be shared on reasonable request to the corresponding author.

## Abbreviations and Acronyms

AR	aortic regurgitation
BAV	bicuspid aortic valve
CI	confidence interval
FET	frozen elephant trunk
IQR	interquartile range
MAE	major adverse events
OM	operative mortality
SCI	spinal cord injury
TAAAD	type A acute aortic dissection
TARP	total arch replacement
